# Evaluation of right ventricular function in fetuses with isolated single umbilical artery using spatiotemporal image correlation M-mode

**DOI:** 10.1186/s12947-019-0164-0

**Published:** 2019-07-20

**Authors:** Tian-gang Li, Fang Nie, Zhen-dong Li, Yan-fang Wang, Qi Li

**Affiliations:** 1 Department of Ultrasound Diagnosis, Lanzhou University Second Hospital, Lanzhou, 730030 Gansu Province People’s Republic of China; 2 Department of Ultrasound Diagnosis, Gansu Provincial Maternity and Child-care Hospital, Lanzhou, 730050 Gansu Province People’s Republic of China

**Keywords:** Fetal heart function, STIC, M-mode, Isolated single umbilical artery, Right ventricular function, F-TAPSE

## Abstract

**Background:**

Postnatal outcome of fetuses with isolated single umbilical artery (SUA) is determined mainly by right ventricular function. Our study examined whether there are differences in right ventricular function during the gestation period of fetuses with isolated SUA compared to healthy fetuses.

**Methods:**

A prospective study was conducted on 77 fetuses with isolated SUA and 77 gestational age-matched controls from 22 to 39 weeks. For gestational age grouping, the isolated SUA fetuses and the control fetuses were divided into the second trimester group (22–27 weeks; 43 fetuses) and third trimester group (28–39 weeks; 34 fetuses). The fetal tricuspid annular plane systolic excursion (f-TAPSE) M-mode was applied to the tricuspid annulus*,* parallel to the ventricular septum, and the amplitude of the resulting wave was assessed using spatiotemporal image correlation (STIC) M-mode. We investigated the possible changes to the STIC M-mode indices during the course of pregnancy in both the isolated SUA and control groups. The relationship between f-TAPSE and gestational age was analyzed. Additionally, the correlations between f-TAPSE and birth weight was analyzed, and the birth weight differences between the isolated SUA and control groups in the third trimester were analyzed according to postpartum results.

**Results:**

There was a significant difference in f-TAPSE between isolated SUA and control group in the third trimester (*P* < 0.05). There were significant correlations between gestational age (GA) and f-TAPSE among control fetuses (R^2^ = 0.9049; *P* < 0.01). A significant, positive correlation between GA and f-TAPSE was also found with isolated SUA fetuses (R^2^ = 0.8108; *P* < 0.01). The prevalence of small-for-gestational-age (SGA) fetuses and of discordant birth weight fetuses was significantly higher in the isolated SUA group than in the control group. In univariate analysis, the presence of an isolated SUA was associated with lower birth weight (2940 g compared with 3260 g) and with higher prevalence of SGA (13.0% compared with 3.9%; *P* < 0.01). The correlations between the birth weight and f-TAPSE in the two groups were analyzed in the third trimester, and the correlation in the isolated SUA group was better than that of the control group (R^2^ was 0.623 and 0.463 in the isolated SUA group and the control group, respectively).

**Conclusions:**

Right ventricular function in isolated SUA is altered as early as in fetal third trimester. STIC M-mode can measure the right heart function of the fetus and may predict isolated SUA with SGA.

## Background

The umbilical cord normally contains two arteries and one vein. The condition characterized by the absence of one of the umbilical arteries (UAs) is referred to as single umbilical artery (SUA) [1, 2]. SUA is one of the most common prenatally diagnosed fetal abnormalities with an incidence of around 0.2–2% [3]. The most widely accepted explanations for this anomaly are primary agenesis or later thrombotic atrophy of the UA. This malformation is 3–4 times more common in multiple pregnancies than in single pregnancies [4]. Approximately 65% of SUA cases appear to be isolated findings [5, 6]. Most cases of isolated SUA are not associated with the presence of other malformations, and in these cases, chromosomal alterations are not usually present. However, more often, isolated SUA cases give rise to the development of certain obstetric complications, such as fetal growth restriction and increased perinatal mortality [7–9]^.^

In pediatric and adult cardiology, the right heart is often targeted to evaluate changes in cardiac function resulting from alterations in preload or afterload, which subsequently affect right heart function [10].Tricuspid annular plane systolic excursion (TAPSE) is a commonly applied measure of right ventricular systolic function that quantifies right ventricular contraction. TAPSE in pediatric and adult patients with heart failure, and is measured by the longitudinal contraction of the right heart; TAPSE is more reliable than shortening fraction, which measures crosswise contraction of the ventricle [11].

The right ventricle functions is the dominant or systemic ventricle, and thus structural or functional anomalies affecting preload or afterload consequently affect fetal performance of the right heart. The objective of this study was to evaluate differences in right ventricular function during gestation between fetuses with isolated SUA and healthy fetuses. Also, we aimed to determine if fetal tricuspid annular plane systolic excursion (f-TAPSE) influences fetal growth and birth weight in cases of isolated SUA.

## Methods

### Study subjects

We prospectively studied 77 fetuses from 22 to 39 weeks of gestational age with prenatally identified isolated SUA and 77 gestational age-matched healthy fetuses in Gansu Provincial Maternity and Child-care Hospital between July 2017 and December 2018. According to gestational age, the isolated SUA fetuses and the healthy fetuses were divided into the second trimester group (22–27 weeks; 43 fetuses) and the third trimester group (28–39 weeks; 34 cases). The study protocol was approved by the hospital ethics committee and pregnant mothers provided their written informed consent. We excluded pregnant mothers with multiple gestations and pregnancies presenting associated fetal anomalies, including structural abnormalities, congenital heart disease, abnormal karyotype, and pregnant mothers with conditions that may affect fetal hemodynamics, such as maternal diabetes, pre-eclampsia, preterm labor, or endocrinological disorders such as thyroid disease.

The diagnosis of isolated SUA was made or confirmed using color Doppler ultrasonography at the level of the fetal abdominal cord insertion, by observing the absence of one of the two umbilical arteries (UAs), which normally encircle the fetal bladder. In all cases, diagnosis of isolated SUA was confirmed by postnatal pathological examination and all newborns were determined to be anatomically normal at delivery. The newborns were diagnosed as small-for-gestational-age (SGA) when birth weight was below the 10th percentile for gestational age. Demographic data including maternal age, weight, height, body mass index, parity, and medical history were collected. Gestational age was calculated based on the first day of the last menstrual period and confirmed by crown–rump length measurement at the first-trimester ultrasound scan.

### Instruments and methods

E10 (GE Healthcare, USA) ultrasound systems were used with the obstetric preset. The Doppler energy was set to < 100 mW/cm^2^. Fetal biometric measurements were performed on each scan. Subsequently, fetal echocardiography was performed by an expert sonographer and the spatiotemporal image correlation (STIC) volume was acquired. In addition to standard biometry, f-TAPSE was also measured in the four-chamber view with the cardiac apex set at the 12 o’clock position. In the STIC M-mode investigation, the M-mode beam was aligned through the lateral aspect of the tricuspid valve annulus, parallel to the interventricular septum.

**Fig. 1:**
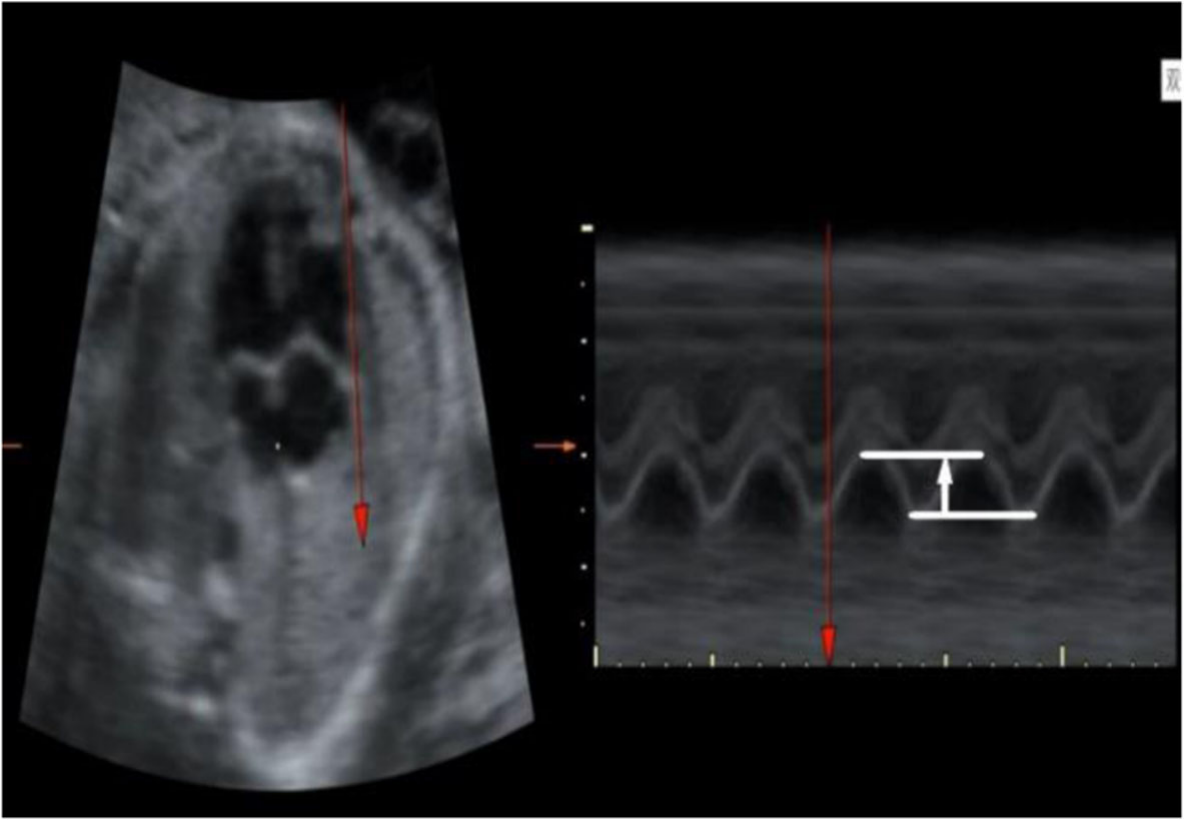
Measurement of spatiotemporal image correlation (STIC) M-mode fetal tricuspid annular plane systolic excursion (f-TAPSE) vertically, parallel to the interventricular septum. Measurement was performed in post-processing and was taken ‘up-up’ on the M-mode trace

The sample box included the whole fetal heart and its adjacent structures. Volume data was acquired when: (1) the probe was held still; (2) no fetal movement was present; (3) at least three complete cardiac cycles were included; (4) the fetus was in a supine position, and the angle between ultrasound beam and the heart was < 30°; (5) there was no or very little shadowing from the ribs in the fetal heart. Qualified images were saved for offline analysis. Measurement on STIC M-mode was then performed with measurement of the amplitude of the wave (Fig. 1). Two to three measures of f-TAPSE were taken and the results were averaged.

### Statistical analysis

Data analysis was performed using IBM SPSS Statistics for Windows, version 23.0 (IBM Corp., Armonk, N.Y., USA). Continuous variables are presented as mean ± SD or median (interquartile range), as appropriate. Linear polynomial regression was used to estimate the relationship between the studied variables and gestational age (GA) expressed in exact weeks (decimal days). Mean and SD curves as a function of GA were calculated and plotted. The equations of the polynomial regression were used to calculate the fitted mean and 5th and 95th centiles for the corresponding GA. Confidence intervals were calculated for the fitted mean. Spearman’s rho was used to assess correlation between f-TAPSE and GA.

## Results

We recruited 154 gravidae for the study, ranging in GA from 22 to 39 (mean, 29 ± 2) weeks. Seventy-seven cases of isolated SUA fetal umbilical cord color flow showed only one umbilical artery and one umbilical vein, including 42 cases of left branch loss and 35 cases of right branch loss; These 77 fetuses showing isolated SUA and 77 normal fetuses were followed up to birth, and basic conditions of neonatal birth between the two groups were analyzed. In univariate analysis, the presence of an isolated SUA was significantly associated with a higher prevalence of SGA (13.0% compared with 3.9%; *P* < 0.01); The differences in body weight and placenta quality between the two groups were statistically significant (*P* < 0.05; Table 1). There were no significant differences in maternal weight, gestational age at delivery, maternal age, and body mass index (*P* > 0.05; Table 1). There was no significant difference in f-TAPSE between isolated SUA group and control group in the second trimester (*P* > 0.05 Table 2). The difference in f-TAPSE between isolated SUA group and control group in the third trimester was statistically significant (*P* < 0.05 Table 2). Isolated SUA and control f-TAPSE were positively correlated with gestational age (*P* < 0.01).

**Table 1:** Demographic and clinical characteristics between the isolated SUA and control group

	isolated SUA group	control group	*P*-value
Maternal weight (kg)	61.9 ± 5.8	61.8 ± 5.5	0.978
Body mass index (kg/m^2^)	24.1 ± 3.6	24.6 ± 3.7	0.853
Maternal age (years)	27.7 ± 5.2	27.5 ± 4.5	0.382
Delivery at week	37.9 ± 1.1	38.8 ± 0.9	0.139
Birth weight (kg)	2.9 ± 0.3	3.3 ± 0.4	0.011
placenta quality (kg)	461 ± 59	523 ± 62	0.000

**Table 2:** Comparison of STIC M-mode f-TAPSE between the isolated SUA and control groups

	Isolated SUA	control	*P*-value
Second trimester group (*n* = 43)	4.46 ± 0.26	4.56 ± 0.26	0.061
Third trimester group (*n* = 34)	5.90 ± 0.97	6.40 ± 1.02	0.042

**Fig. 2:**
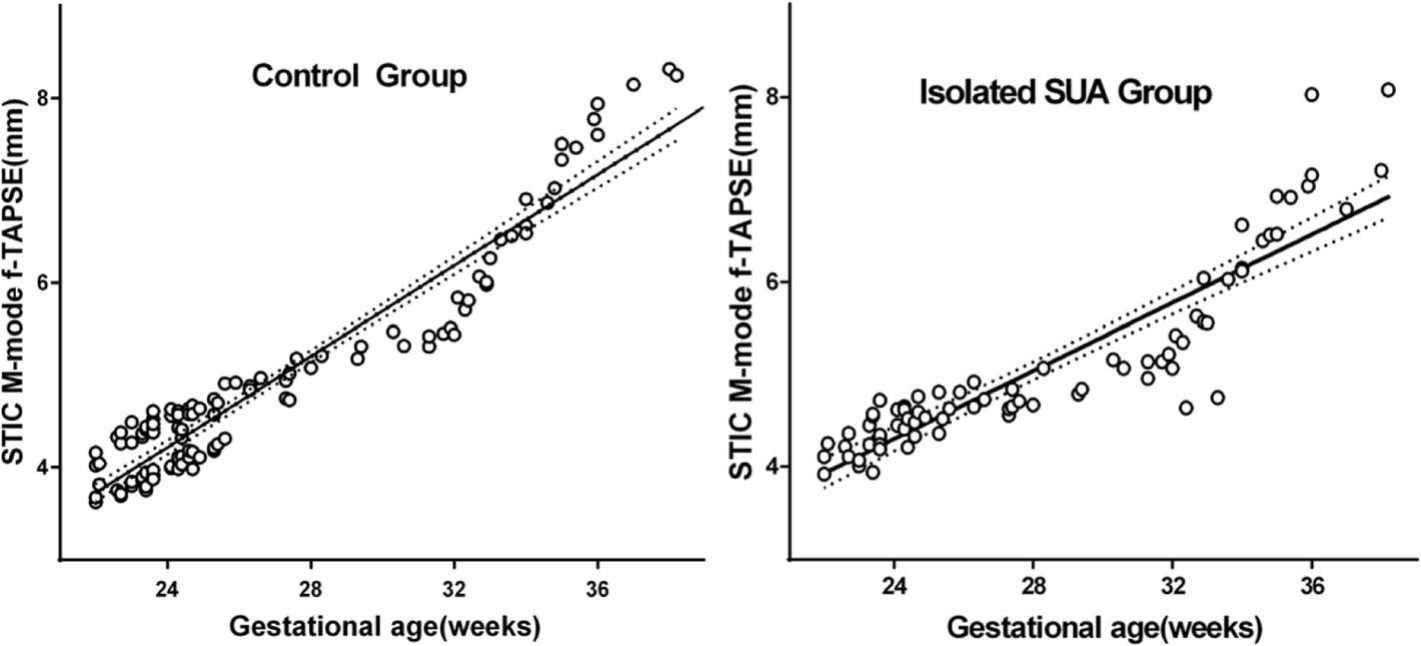
Scatterplots, with isolated SUA, of spatiotemporal image correlation (STIC) M-mode fetal tricuspid annular plane systolic excursion (f-TAPSE) against: (Control) estimated f-TAPSE (regression line: 0.2292 × GA − 1.112 R^2^ = 0.90); and (isolated SUA) estimated f-TAPSE (regression line: 0.1847 × GA − 0.1341 R^2^ = 0.81)

**Fig. 3:**
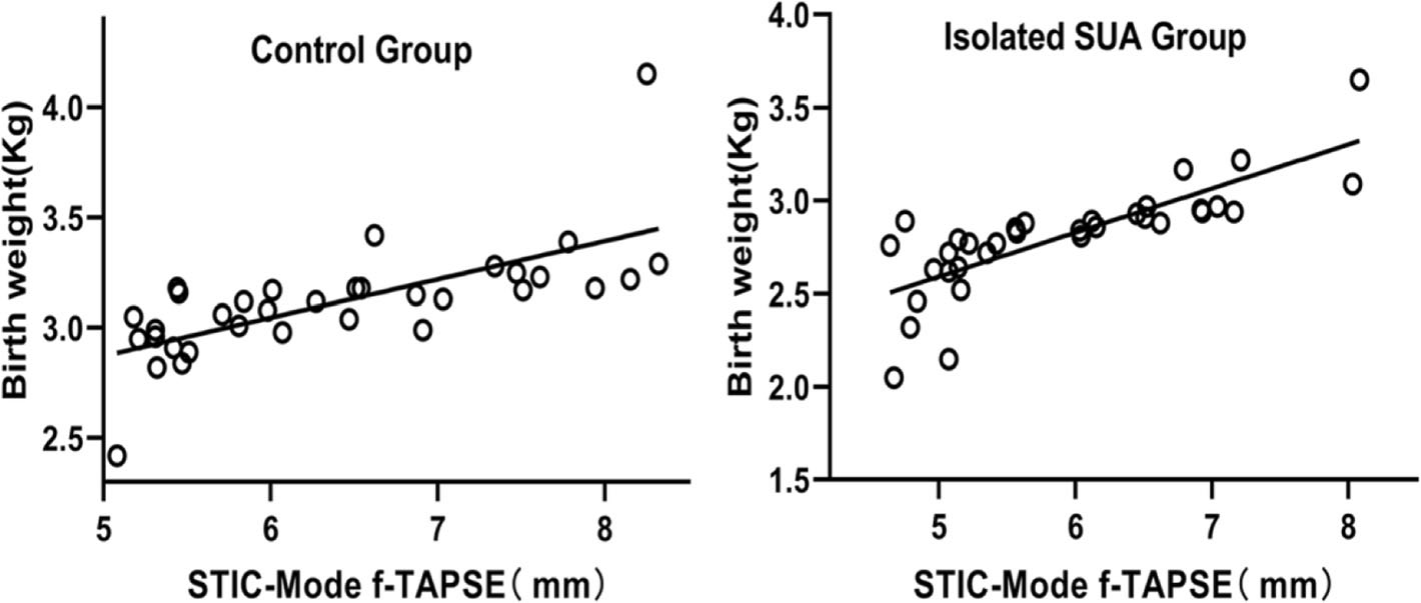
Scatterplots, with isolated SUA, of the correlations between the birth weight and spatiotemporal image correlation (STIC) M-mode fetal tricuspid annular plane systolic excursion (f-TAPSE) against: (Control) estimated birth weight (regression line:0.1734 × f-TAPSE+ 2.006 R^2^ = 0.4630); and (isolated SUA) estimated birth weight (regression line: Y = 0.2371 × f-TAPSE + 1.407 R^2^ = 0.623)

Statistical software analysis showed that GA and f-TAPSE had a high positive correlation. The scatter plot in Fig. 2 showed a linear correlation between GA and f-TAPSE, the correlation was good, and all were positively correlated (*P* < 0.001). The linear regression equation of normal fetal f-TAPSE and GA is: Y = 0.2292 × GA − 1.112 (R^2^ = 0.90) and the linear regression equation of isolated SUA fetal f-TAPSE and GA is: Y = 0.1847 × GA − 0.1341 (R^2^ = 0.81). The correlations between the birth weight and f-TAPSE in the two groups in the the third trimester were analyzed, and the correlation in the isolated SUA Group was better than that of the control group (R^2^ was 0.623 and 0.463 in the isolated SUA group and the control group, respectively) (Fig. 3).

## Discussion

We found no evidence that fetuses with isolated SUA have increased risk of aneuploidy. Fetuses with an apparently isolated SUA potentially have increased risk of impaired fetal growth. In this study we confirmed that the presence of an isolated SUA is associated with SGA. The finding of an isolated SUA being associated with SGA birth has been shown previously; it may not be related directly to the composition of the umbilical cord itself, but rather to a wider pathological process affecting the placenta [12]. The presence of an isolated SUA is associated with SGA, pregnancy-induced hypertension, and medically indicated preterm birth [3]. However, some other studies have shown that certain obstetric complications were often observed in isolated SUA cases, such as fetal growth restriction and increased perinatal mortality [13].

In addition to detecting abnormal fetal heart structure, ultrasound can also quantitatively evaluate fetal heart hemodynamics and cardiac function. There are many methods for evaluating fetal cardiac function, including common M-mode, atrioventricular valve blood flow velocity measurement, TEI index, and myocardial strain–strain rate analysis. Each of these methods performs a cardiac evaluation from a slightly different angle, applying different techniques that have their own advantages. The right ventricle is the dominant or systemic ventricle in the fetus, but it is somewhat less studied than the left ventricle. The left ventricle can be evaluated very effectively with the modified myocardial performance index (mod-MPI), but this measure is cumbersome in the right ventricle; the two Doppler measures necessary for MPI calculation must be made in two separate planes in the right heart, so they cannot be obtained during the same cardiac cycle [14, 15]. Other methods of evaluation of right heart function have been applied in fetuses or are common in children and adults. Despite their advantages, these methods have their own disadvantages. Systolic function assessment also includes measurement of pulmonary artery peak velocities, TAPSE, 3D/4D ultrasound-based measures, tissue Doppler derived measures, speckle tracking, and magnetic resonance imaging (MRI).

The STIC technology closely links fetal heart three-dimensional data with time information and can directly perform three-dimensional dynamic ultrasound imaging of fetal heart. It can directly measure the right ventricular systolic function of the fetus by directly measuring the amplitude of the tricuspid annulus. f-TAPSE is a commonly used measure of right ventricular systolic function, and the biggest advantage of this method is that the sound beam is perpendicular to the annulus to reduce the measurement error caused by the ultrasonic beam angle, which improves the accuracy and repeatability of the measurement. In addition, we were able to return to saved volumes to examine the f-TAPSE any time after the examination. Thus, this evaluation of the fetal heart function is more convenient and accurate. We used STIC M-mode interrogation firstly for evaluation of right ventricular myocardial function in fetuses with isolated SUA and in those with normal cardiac anatomy. Secondly, we aimed to test for possible changes in right ventricular function with gestational age. According to our protocol, fetuses of ≥22 weeks of gestational age were included in this study, because feasibility and reproducibility of STIC M-mode during the first trimester of gestation remains technically challenging.

Many studies have evaluated fetal heart function mainly focusing on pregnancy complications, which is more common in pregnancy-induced hypertension and gestational diabetes. SUA is a common obstetric complication in clinical practice, and because in SUA fetuses lack an umbilical artery, it may cause fetal hemodynamic changes and consequently affect fetal cardiac function [9]. However, current reports and research on cardiac function of fetuses having an isolated SUA are not common, and thus we lack a common understanding of this condition. At present, f-TAPSE has been recognized as a sensitive index to assess right heart function and is widely used in the assessment of right heart systolic function in children and adults [16, 17]. But unfortunately, there are few reports on the evaluation of fetal right heart function by f-TAPSE application. This study aims to accurately assess right ventricular systolic function of isolated SUA using the STIC-M technique, which can better assess intrauterine development and outcome of isolated SUA, and thus provide an objective basis for mother and child monitoring during pregnancy.

We found no significant difference in f-TAPSE of the isolated SUA between the second trimester group and the control group, indicating that the right systolic function of the isolated SUA in the second trimester group has not changed significantly. The first possible explanation is: although fetuses with isolated SUA have one less umbilical artery than healthy fetuses, the fetal isolated SUA may increase its inner diameter to compensate for the reduced blood flow, thus ensuring adequate cardiac output to the fetus [18, 19]. Previous studies have demonstrated the ability of the SUA to compensate with gradual dilation to decrease resistance and carry twice the blood volume of an artery in a three-vessel cord [19]. The second possibility is the early hemodynamic changes in the second trimester in fetuses with isolated SUA. This stage is not enough to cause changes in the right heart contractility and function of the fetus. Finally, fetal hemodynamics may change due to the lack of an UA in the isolated SUA fetus. The fetus conducts its own blood flow regulation through the “brain protection effect” to achieve the balance of overall blood flow.

In this study, the difference of f-TAPSE between isolated SUA and normal group in the third trimester group was significant, indicating that the right systolic function of the isolated SUA in the third trimester was lower than that in the normal group. So we should suggest to do the ‘high risk follow-up’ only at the third trimester with isolated SUA fetuses. Possible reasons for this include: First, the incidence of preterm birth and low body weight in isolated SUA is obvious. The frequency of SGA was higher among pregnancies complicated by an isolated single umbilical artery higher than control fetuses, as we observed low fetal weight in isolated SUA group compared to control. Perhaps the isolated SUA fetus is more likely to have a lower birth weight and f-TAPSE than control group. Cardiovascular changes persist into childhood in the form of remodeled and less efficient globular hearts, hypertension, and increased vascular wall thicknes [20]. Several studies have demonstrated that SGA fetuses present subclinical signs of cardiac dysfunction [21, 22]. Nevertheless, even in structurally and karyotypically normal fetuses, the finding of an isolated SUA has been associated with SGA, which may not be related directly to the composition of the umbilical cord itself, but rather to a wider pathological process. Secondly, changes in fetal right heart function during late pregnancy may be caused by changes in isolated SUA fetal placental structure and blood flow. In this study, the fetal placental quality of the isolated SUA group was lower than that of the control group. It might be that the isolated SUA fetal placental structure and blood flow changes pose a greater risk for the isolated SUA fetus to be born with low birth weight and lead to a decrease in right systolic function. Some studies have shown that fetuses with isolated SUA are at greater risk of abnormal placental development and perfusion rather than inadequate fetoplacental blood flow through the remaining umbilical artery [12].

We also showed that isolated SUA and normal f-TAPSE were positively correlated with gestational age, indicating that isolated SUA and normal fetal contractile function were correlated with fetal gestational age. This may increase with fetal gestational age and with increased fetal weight. Greater blood capacity is required to provide nutrients for themselves, and the correlation between isolated SUA f-TAPSE and GA is lower than in the control group. This might be due to the low correlation between f-TAPSE and GA secondary to the low birth weight of isolated SUA fetuses. Finally, the correlations between the birth weight and f-TAPSE in the two groups of third trimester were analyzed, and the correlation in the isolated SUA group was better than that of the control group. Previous study have showed that f-TAPSE had a steady linear increase over the course of gestation and strong correlation with GA and estimated fetal weight [14]. So, the effect that causes f-TAPSE between the two groups may be due to the difference in fetal weight. So, the authors believe that if the study continues in the future with a large sample size of the isolated SUA, we can predict the weight of isolated SUA fetuses with f-TAPSE in third trimester.

## Conclusions

STIC technology can be used to detect the right cardiac function of isolated SUA and determine changes in cardiac systolic function. STIC can also help to make an objective intrauterine assessment of the occurrence and development of isolated SUA, predict prognosis, and guide clinical testing.

## Data Availability

The data and material in the current study are available from the corresponding author on reasonable request.

## Abbreviations

f-TAPSE: fetal tricuspid annular plane systolic excursion
SGA: small for gestational age
STIC: spatiotemporal image correlation
SUA: single umbilical artery
UAs: umbilical arteries

## References

[1] Araujo Júnior E1, Palma-Dias R, Martins WP, et al. Congenital heart disease and adverse perinatal outcome in fetuses with confirmed isolated single functioning umbilical artery [J]. J Obstet Gynecol 2014; 14: 1–3. 10.3109/01443615.2014.935720 25020205

[2] Bugatto F, Quintero-Prado R, Melero-Jiménez V (2010). Ultrasound pre-dictors of birth weight in euploid fetuses with isolated sisingleum-bilical artery. Ultrasound Obstet Gynecol.

[3] Gornall AS, Kurinczuk JJ, Konje JC (2003). Antenatal detection of a single umbilical artery: does it matter?. Prenat Diagn.

[4] Volpe G, Volpe P, Boscia FM, Volpe N, Buonadonna AL, Gentile M (2005). Isolated’ single umbilical artery: incidence, cytogenetic abnormalities, malformation and perinatal outcome. Minerva Ginecol.

[5] Voskamp BJ, Fleurke-Rozema H, Oude-Rengerink K (2013). Relationship of isolated single umbilical artery to fetal growth, aneuploidy and perinatal mortality; systematic review and meta-analysis. Ultrasound Obstet Gynecol.

[6] Granese R, Coco C, Jeanty P (2007). The value of single umbilical artery in the prediction of fetal aneuploidy: findings in 12,672 pregnant women. Ultrasound Q.

[7] De Catte L, Burrini D, Mares C, Waterschoot T (1996). Single umbilical artery: analysis of Doppler flow indices and arterial diameters in normal and small-for-gestational age fetuses. Ultrasound Obstet Gynecol.

[8] Martínez-Payo C, Gaitero A, Tamarit I, García-Espantaleón M, Iglesias Goy E (2005). Perinatal results following the prenatal ultrasound diagnosis of single umbilical artery. Acta Obstet Gynecol Scand.

[9] Horton AL, Barroilhet L, Wolfe HM (2010). Perinatal outcomes in isolated single umbilical artery. Am J Perinatol.

[10] Lopez-Candales A, Rajagopalan N, Saxena N, Gulyasy B, Edelman K, Bazaz R (2006). Right ventricular systolic function is not the sole determinant of tricuspid annular motion. Am J Cardiol.

[11] Koestenberger M, Nagel B, Ravekes W, Everett AD, Stueger HP, Heinzl B, Sorantin E, Cvirn G, Gamillscheg A (2010). Tricuspid annular plane systolic excursion and right ventricular ejection fraction in pediatric and adolescent patients with tetralogy of Fallot, patients with atrial septal defect, and age-matched normal subjects. Clin Res Cardiol.

[12] Murphy-Kaulbeck L, Dodds L, Joseph KS, Van den Hof M (2010). Single umbilical artery risk factors and pregnancy outcomes. Obstet Gynecol.

[13] Hua Meiling, Odibo Anthony O., Macones George A., Roehl Kimberly A., Crane James P., Cahill Alison G. (2010). Single Umbilical Artery and Its Associated Findings. Obstetrics & Gynecology.

[14] Messing B, Gilboa Y, Lipschuetz M, Valsky DV, Cohen SM, Yagel S (2013). Fetal tricuspid annular plane systolic excursion (f-TAPSE): evaluation of fetal right heart systolic function with conventional M-mode ultrasound and spatiotemporal image correlation (STIC) M-mode. Ultrasound Obstet Gynecol.

[15] Hernandez-Andrade E, Benavides-Serralde JA, Cruz-Martinez R, Welsh A, MancillaRamirez J (2012). Evaluation of conventional Doppler fetal cardiac function parameters: E/a ratios, outflow tracts, and myocardial performance index. Fetal Diagn Ther.

[16] Park JH, Kim JH, Lee JH, Choi SW, Jeong JO, Seong IW (2012). Evaluation of right ventricular systolic function by the analysis of tricuspid annular motion in patients with acute pulmonary embolism. J Cardiovasc Ultrasound.

[17] Zakeri SA, Panayotova R, Borg AN (2014). Cardiovascular magnetic resonance validation of fractional changes in annulo-apical angles and tricuspid annular plane systolic excursion for rapid as sessment of right ventricular systolic function. J Magn Reson Imaging.

[18] De Catte L, Burrini D, Mares C, Waterschoot T (1996). Single umbilical artery: analysis of Doppler flow indices and arterial diameters in normal and small-for-gestational age fetuses. Ultrasound Obstet Gynecol.

[19] Goldkrand JW, Pettigrew C, Lentz SU, Clements SP, Bryant JL, Hodges J (2001). Volumetric umbilical artery blood flow: comparison of the normal versus the single umbilical artery cord. J Matern Fetal Med.

[20] Crispi F, Bijnens B, Figueras F, Bartrons J, Eixarch E, LeNoble F, Ahmed A, Gratacós E (2010). Fetal growth restriction results in remodeled and less efficient hearts in children. Circulation.

[21] Comas M, Crispi F, Cruz-Martinez R, Figueras F, Gratacos E (2011). Tissue Doppler echocardiographic markers of cardiac dysfunction in small-for-gestational age fetuses. Am J Obstet Gynecol.

[22] Cruz-Lemini M, Crispi F, Valenzuela-Alcaraz B, Figueras F, Sitges M, Gómez O, Bijnens B, Gratacós E (2013). Value of annular M-mode displacement vs tissue Doppler velocities to assess cardiac function in intrauterine growth restriction. Ultrasound Obstet Gynecol.

